# Evaluation of Respiratory Muscular Strength Compared to Predicted Values in Patients with Stroke

**DOI:** 10.3390/ijerph17031091

**Published:** 2020-02-09

**Authors:** Sarah Maria Ramos, Daniela Maciel da Silva, Daniela Vieira Buchaim, Rogério Leone Buchaim, Mauro Audi

**Affiliations:** 1 Faculty of Physiotherapy, University of Marilia (UNIMAR), Avenue Hygino Muzzy Filho, 1001, Marília 17525-902, São Paulo, Brazil; sarah.ramos1@hotmail.com (S.M.R.); maciel.daniela@hotmail.com (D.M.d.S.); mauroaudi@unimar.br (M.A.); 2 Postgraduate Program in Structural and Functional Interactions in Rehabilitation, University of Marilia (UNIMAR), Avenue Hygino Muzzy Filho, 1001, Marília 17525-902, São Paulo, Brazil; danibuchaim@usp.br; 3 Medical School, University Center of Adamantina (UniFAI), Nove de Julho Street, 730–Centro, Adamantina 17800-000, São Paulo, Brazil; 4 Department of Biological Sciences (Anatomy), Bauru School of Dentistry, University of São Paulo (USP), Alameda Dr. Octávio Pinheiro Brisolla, 9-75–Vila Universitaria, Bauru 17012-901, São Paulo, Brazil

**Keywords:** functional rehabilitation, maximal respiratory pressure, muscle strength, physiotherapy, public health, quality life, stroke

## Abstract

The purpose of this study was to evaluate the inspiratory and expiratory muscle strength of individuals affected by stroke and to compare it with the predicted values in the literature considering their corresponding age. Respiratory muscle strength was evaluated in 22 elderly people who had sequels of stroke, four with right hemiparesis, 16 with left hemiparesis and two with bilateral, of ages ranging from 34 to 82 years. The collected data were submitted to statistical analysis using a Mann–Whitney test to evaluate if there was a significant difference in the average data collected when compared with a mean of the predicted data in the literature. Fourteen men and eight women were evaluated, who obtained mean values of 71.85 cmH_2_O and 57.75 cmH_2_O, respectively, for a maximal inspiratory pressure (MIP), and when compared to the predicted values for men and women, 105.41 cmH_2_O (*p*-value 0.0019) and 80.57 cmH_2_O (*p*-value 0.00464) were significantly lower. For a maximal expiratory pressure (MEP), the mean value obtained for men was 62.28 cmH_2_O and 49.5 cmH_2_O for women, whereas the predicted values in the literature were 114.79 cmH_2_O (*p*-value < 0.0001) and 78, 46 cmH_2_O (*p*-value 0.0059), respectively. In the statistical analysis, it was possible to notice that the studied population did not reach the predicted age indexes and that there was a significant difference between the median columns. In conclusion, there is a weakness in the respiratory muscles of hemiparetic men and women due to stroke.

## 1. Introduction

Stroke is clinically defined as an acute neurological dysfunction of vascular origin and is classified as the third most fatal disease in several countries worldwide [1,2]. In addition, it can be explained as a vascular alteration that acutely or intermittently weakens blood flow in the brain, caused by arterial obstruction (ischemic stroke) or its rupture (hemorrhagic stroke) [3]. The clinical signs are directly related to the location and extent where the injury occurred [4].

The nervous system, when affected by a pathology, can cause a physical and mental disability that, consequently, leads to some sequelae, such as paralysis, sensory and muscle tone changes, general biomechanical movement and breathing pattern. The paresis found in the compromised hemibody, which consequently can affect the diaphragm muscle and possible postural deviations in the trunk, can trigger respiratory changes in these individuals [5].

When these changes are associated with clinical manifestations underlying the stroke, the respiratory muscle strength of these individuals can be severely affected. Thus, changes in motor activity may occur on an affected side [6].

In addition to this fact, an inability to perform daily activities and tasks can develop due to biomechanical changes in the respiratory system, as it involves the complex interaction between inspiratory and expiratory muscles, the rib cage and abdomen, causing impairment of lung function and increasing the risk of lung infections due to expiratory muscle weakness and cough inefficiency [7]. 

Because of a stroke, there is a severe loss of muscle tone and a decrease in aerobic capacity, which consequently can lead to an increase in energy expenditure in daily activities, especially in hemiplegic and elderly patients. This energy expenditure can increase up to two times, causing fatigue and muscle weakness [8].

The assessment of respiratory muscle strength should not be restricted to patients with lung diseases, but should also be performed in patients with neuromuscular diseases, as they are very susceptible to the development of skeletal and respiratory muscle weakness. In addition, biomechanical disorders can cause respiratory complications [9,10].

Due to the scarcity of studies that contextualize regions of Brazil and, consequently, South America, that analyze the association of stroke and respiratory capacity, which can guide the process of motor recovery through physiotherapy, this study was proposed with the objective of evaluating the inspiratory and expiratory muscle strength of individuals affected by stroke and compare it with the values provided in the literature considering the corresponding age.

## 2. Materials and Methods 

### 2.1. Clinical Study Design

All subjects gave their informed consent for inclusion before they participated in the study. The study was conducted in accordance with the Declaration of Helsinki, and the protocol (number 2.083.545) was approved by the Research Ethics Committee of the University of Marilia (UNIMAR, Marília, SP, Brazil), according to resolution 466/12 of the National Health Council of Brazil. The clinical study was conducted at the Clinic of the Faculty of Physiotherapy of the University of Marília -UNIMAR.

The participants in this research, who accepted to participate voluntarily, were selected according to the following formulated criteria:

- Inclusion criteria:
  Diagnosis of previous stroke; presentation of the referral term of the medical professional to the Physiotherapy clinic of the University of Marília; over 18 years of age; patients of the Brazilian Unified Health System (SUS) in the cities covered by the Regional Health Directorate of Marília (DRS IX).
- Exclusion criteria:
  Absence of medical referral with diagnosis of stroke; under 18 years old; other previous comorbidities such as dementia, depression, atrial fibrillation or previous respiratory diseases; use of medications such as warfarin; patients in private health systems; patients outside the scope of DRS IX—Marília.

Twenty-two individuals volunteered and signed an informed consent form (ICF). This group was composed of patients aged between 34 and 82 years (8 women and 14 men), who presented sequelae following stroke. Four individuals who volunteered in this study were patients with right hemiparesis, 16 with left hemiparesis and 2 with bilateral, all of whom were in the rehabilitation phase.

Participating in this study were conscious and collaborative individuals with hemiparesis due to stroke, with the ability to grasp the lips, without apparent chest deformity and with the ability to remain in the sitting position.

The time stipulated in this study for obtaining the sample, through the referrals mentioned above, occurred for a period of 60 days. The rehabilitation process with the attendance at the physiotherapy clinic lasted approximately 180 days.

The recruitment was completed by two researchers who were trained in such a way as to obtain a level of maximal inspiratory pressure (MIP) and maximal expiratory pressure (MEP) collection, always performed in the same place, one of the University’s Physical Therapy clinical rooms.

The preference of the materials used has always been for disposables. For equipment in common use, the surfaces were decontaminated using rubbing alcohol 70% (w/v). The vacuum manometer device was calibrated before the beginning of the study according to recommendations by INMETRO (National Institute of Metrology, Quality and Technology of Brazil).

### 2.2. Procedures

Measurements of MIP and MEP were performed using a vacuum manometer (Indumed^®^, São Paulo, Brazil) with an operating range of −120 cmH_2_O to +120 cmH_2_O, which was connected to a plastic trachea 30 cm long and 2.4 mm of internal diameter. The end portion of the trachea was connected to a rigid plastic connection.

For the measurements of respiratory muscle strength, we followed the protocol cited by Parreira et al. [11], that indicates that maximal respiratory pressure measurements should be performed with the individuals seated, using a nasal clip and placing the buccal of the vacuum manometer firmly between the lips. Two maneuvers for learning were performed, which were considered acceptable maneuvers that prevented air leaks and where pressure could be maintained for at least one second. There was an interval of one minute between the three measurements, and the highest value between reproducible maneuvers was the one selected for analysis.

For the MIP measure, individuals should exhale up to residual volume which, subsequently, generates a maximal inspiratory effort against an occluded airway. For MEP, individuals should inhale up to the total lung capacity, and then a maximal expiratory effort against an occluded airway will be generated [11]. According to the values found in the evaluation, the inspiratory (MIP) and expiratory (MEP) strength values were compared with the values predicted in the equations [12]:

MIP—Women: y = −0.49 (age) + 110.4 —Man: y = −0.80 (age) + 155.3 MEP—Women: y = −0.61 (age) + 115.6 —Man: y = −0.81 (age) + 165.3

Negative values displayed in the vacuum manometer during the test refers to the inspiratory pressure and the positive values to the expiratory pressure. To inspiratory muscles, normal values range from −75 to −120 cmH_2_O and lower indexes indicate degrees of weakness (−70 to −45 cmH_2_O), fatigue (−40 to −25 cmH_2_O) and failure (less than or equal to −20 cmH_2_O). For expiratory muscles, the normal values are between +100 and +120 cmH_2_O, and weakness is characterized as being when values are below +95 cmH_2_O [13].

### 2.3. Data Analysis

For the statistical analysis of collected data, a tabulation was performed, splitting the subjects into four categories, which referred to the values of MIP and MEP for men and women.

Data were transferred to the BioEstat 5.3 Software (Mamirauá Institute, Manaus, Brazil), to compare the median columns values of maximum respiratory pressures attained and the predicted values using the Mann–Whitney test.

## 3. Results

Values of maximum respiratory pressures in patients with stroke were evaluated considering a divide group of men and women in order to correspond it with the predicted indexes.

Results from the parameters evaluated were tabulated in an Excel spreadsheet and a median columns analysis was performed. The responses of MIP for the 14 men was 71.85 cmH_2_O and MEP of 62.28 cmH_2_O, while for women MIP was 57.75 cmH_2_O and MEP of 49.50 cmH_2_O.

**Figure 1:**
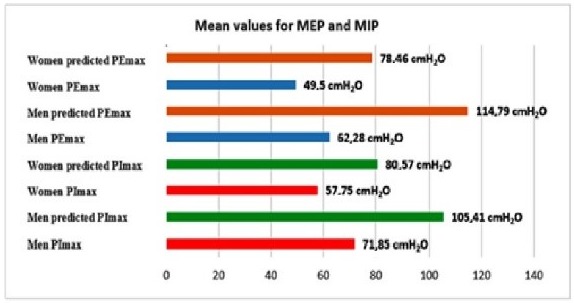
Comparison of values for maximal inspiratory pressure (MIP) and maximal expiratory pressure (MEP) from the assessed group compared to the predicted values on literature.

The collected data were compared to the values found in literature according to individuals of similar age and gender, and the results revealed predicted values of MIP for men of 105.41 cmH_2_O and MEP of 114.79 cmH_2_O while the MIP predicted values for women were 80.57 cmH_2_O and MEP of 78.46 cmH_2_O, as shown in Figure 1.

It should be highlighted that median values were used for the statistical calculations, however, in an individual analysis, three participants presented values of MIP above the predicted values, with values of 100; 104 and 120 cmH_2_O, and their respective predicted value would have been 93.74; 79.04 and 100.9 cmH_2_O. The MEP values were in accordance with the literature.

Regarding to the MIP of hemiparetic patients (14 men with average age of 62.3 ± 11.3 years) the average value was 66.0 cmH_2_O and the predicted value for this parameter was 106.0 cmH_2_O. Analysis of the results through Mann–Whitney test reported a significant (*p* < 0.0019) reduction in maximal inspiratory pressure in the individuals evaluated (Table 1). Eight women with an average age of 60.8 ± 11.5 years were evaluated regarding MIP, and it was revealed an average value of 47.0 cmH_2_O while the predicted mean value was 78.0 cmH_2_O (*p* < 0.0464), as shown in Table 1.

The median value for expiratory pressure (MEP) of the 14 men assessed was 54.0 cmH_2_O, and when it compared to the predicted values on literature (115.5 cmH_2_O) it is noticed that the value attained on this study was significantly lower (*p* < 0.0001) as seen in Table 2. From the eight women evaluated, the mean value identified for MEP was 42.0 cmH_2_O, while the mean value predicted was 75.0 cmH_2_O, which resulted in a significant decrease in MEP for women with stroke (*p* < 0.0059).

The *p*-value found in all analyses was lower than the value of statistical significance, which is 0.05, thus showing that there is a significant difference between the values collected and the predicted values, which corresponds to a reduction in MIP and MEP values for both groups after stroke.

Regarding the body height of the participants in this study, the values of 1.71 ± 0.13m were obtained for men and for women 1.64 ± 0.11m (mean ± standard deviation). Regarding body weight, the values of 74.7 ± 8.3 kg for men and 63.2 ± 7.4 kg for women were obtained.

## 4. Discussion

The main objective of this study was to evaluate the inspiratory and expiratory muscle strength of stroke patients and compare with the values predicted in the literature considering the corresponding age. It was observed that stroke patients had respiratory muscle weakness, regardless of gender and age. The average age was 62.3 ± 11.3 for men and 60.8 ± 11.5 for women. Several factors contribute to decrease the strength of the respiratory muscles. The patients observed were directly related to a pathology, stroke, which results in altered motor function, a fact that probably contributed to the results presented, since the muscles of the affected side have paresis and physical disability [5,14].

In this study, mean MIP and MEP values were identified for men and women, suggesting a weakness in the respiratory muscles. Similar studies evaluating stroke patients exhibiting MIP and MEP values were lower when compared to Brazilian literature [5,7]. Moreover, besides the disease other factors may decrease the strength of the respiratory muscles, usually the sequalae patients due to stroke, greatly reduce daily activities, which makes them sedentary. Even before the pathology, they already display lack of physical activity, cardiovascular pathology and respiratory weakness [15,16]. The respiratory muscles strength can also be determined by age, gender, muscle development, strength–speed ratio and frequency–velocity ratio [17].

Physical inactivity is an important factor and plays an important role in determining lower muscle mass and higher prevalence of physical disability. It was found in this study that one of the patients (68 years) was a police officer, and his performance in relation to MIP was above expectations for age. This can be explained by the fact that, when young, this patient performed activities that may develop strength in the body and, therefore, part of his muscular strength was preserved. This is in line with the literature, where it has been reported that resistance exercise from childhood is beneficial in preventing muscle loss as the individual ages, making it an important strategy for maintaining and increasing muscular mass and strength [18,19].

Regarding gender, the results of this study showed that females had a lower mean value for MIP than that noticed for men, and this specific behavior is in agreement with several authors who verified a higher index of MIP in men when compared to women [20].

Data found in the literature—stating that muscle loss is directly related to aging, and that muscle loss occurs, in part, due to a significant decline in the number and size of type I and II muscle fibers—suggest that, as a consequence, there may also be a decrease in ventilation mechanics [21]. Younger patients usually do not have number and size of muscle fibers, which may be justified, as the 34-year-old patient has a normal range related to the predicted age. Nonetheless, considering the average age around of 60 years, the reduction in strength of the respiratory muscles is justified.

Studies evaluating muscle respiratory strength in sedentary and physically active elderly women [22,23], verified by MIP and MEP, were higher in the participants—a fact also observed in the literature where it was related that physical exercises in general, even if not oriented to the respiratory muscles and/or pulmonary function, resulted in better respiratory muscle performance [22,24,25]. This allows us to explain in our study that the 64-year-old patient, who regularly practices short walks, reaches the predicted values according to age.

In a study of hemiparetic patients, in which 17 were in the acute phase and 12 in the chronic phase, it was found that patients in the acute phase showed higher mean values for MIP than in the chronic phase [7]. This fact was not noticed in this study, as there were only two participants in the acute phase, so it was impossible to perform a reliable statistical analysis.

The values of MIP and MEP of all the evaluated patients did not reach the predicted indices described in the Brazilian literature according to age. Given this fact, it can be suspected that the impairment of respiratory muscles may be related to the occurrence of a stroke [26,27].

Because many diseases can affect the reliability of the results of this study, patients also suffering from clinical situations involving dementia, depression, atrial fibrillation, previous respiratory diseases—which are very common comorbidities—were excluded from the research. In addition, treatment with warfarin or any other treatment that may have also affected the results was checked and the participants were excluded [28,29].

The number of research participants can be considered a limitation of this study, mainly due to the inclusion and exclusion criteria. Patients with other morbidities could compromise the real objectives of the research, as well as the veracity of the results.

Finally, bearing in mind that the real knowledge of respiratory capacity in stroke patients provides better conditions for the patient’s rehabilitation, and that respiratory improvement can contribute to motor recovery in general, favoring the prognosis, there are future perspectives new studies to assess the final gain in patients’ quality of life [30,31,32,33,34,35,36,37].

## 5. Conclusions

It was possible to conclude that, under the conditions in which this study was carried out, muscle weakness was observed in men and women with hemiparetics. Therefore, it is very important to suggest activities that help to improve respiratory muscle strength after a stroke, to avoid further complications, to provide conditions for functional recovery and, consequently, to improve quality of life. 

## Figures and Tables

**Table 1:** Median values for maximum inspiratory pressure (MIP) in men and women.

	Men	(MIP)	Women	(MIP)
Results	Predicted Values	Collected Values	Predicted Values	Collected Values
Sample Size	14	14	8	8
Sum of Posts (Ri)	266.0	140.0	84.0	52.0
Median	106.0	66.0	78.0	47.0
U	35.0		16.0	
Z (U)	2.8947		1.6803	
*p*-value (unilateral)	0.0019		0.0464	
*p*-value (bilateral)	0.0038		0.0929	

The values in the column 1 refers to the predicted values and in the column 2 the attained values on this research through the Mann–Whitney test. Source: *BioEstat*. Significant difference (*p* < 0.05).

**Table 2:** Median values for maximum expiratory pressure (MEP) in men and women.

	Men	(MEP)	Women	(MEP)
Results	Predicted Values	Collected Values	Predicted Values	Collected Values
Sample Size	14	14	8	8
Sum of Posts (Ri)	293.0	113.0	92.0	44.0
Median	115.5	54.0	75.0	42.0
U	8.00		8.0	
Z (U)	4.1353		2.5205	
*p*-value (unilateral)	< 0.0001		0.0059	
*p*-value (bilateral)	< 0.0001		0.0117	

The values in the column 1 refers to the predicted values and in the column 2 the attained values on this research through the Mann–Whitney test. Source: *BioEstat*. Significant difference (*p* < 0.05).
